# Interference and Inhibition in Bilingual Language Comprehension: Evidence from Polish-English Interlingual Homographs

**DOI:** 10.1371/journal.pone.0151430

**Published:** 2016-03-15

**Authors:** Joanna Durlik, Jakub Szewczyk, Marek Muszyński, Zofia Wodniecka

**Affiliations:** 1 Institute of Psychology, Jagiellonian University, Krakow, Poland; 2 The Pennsylvania State University, University Park, Pennsylvania, United States of America; Leiden University, NETHERLANDS

## Abstract

The main goal of the present study was to explore the involvement of inhibition in resolution of cross-language activation in bilingual comprehension and a possible modulatory effect of L2 proficiency. We used a semantic relatedness judgment task in L2 English that included Polish-English interlingual homographs and English translations of the Polish homographs’ meanings. Based on previous studies using the same paradigm, we expected a strong homograph interference and inhibition of the homographs’ Polish meanings translations. In addition, we predicted that participants with lower L2 proficiency would experience greater interference and stronger inhibitory effects. The reported results confirm a strong homograph interference effect. In addition, our results indicate that the scope of inhibition generalized from the homograph’s irrelevant meaning to a whole semantic category, indicating the flexibility of the inhibitory mechanisms. Contrary to our expectations, L2 proficiency did not modulate the effects of interference and inhibition, possibly due to a relatively low variability in proficiency within our participant sample.

## Introduction

Empirical evidence shows that when a bilingual recognizes a word, there is parallel (*non-selective*) co-activation of lexical entries from both languages (see [1–2] for reviews). The effects of non-selectivity in lexical processing have been observed in studies employing words that share both orthography and semantics (*cognates*) [3], share phonology or orthography but not meaning (interlingual *homophones* or *homographs*, also known as *false friends*) as well as cross-language orthographic or phonological neighbors which have some overlap in orthography or phonology [4]. Interestingly, it seems that even sentence context (i.e. language of preceding words in a sentence) is not always a sufficient cue to limit the activation of an irrelevant meaning of an ambiguous word [2]. For example, when a Polish-English bilingual reads the sentence "Yesterday, she baked two *pies*", the cross-linguistically ambiguous homographic word *pies* activates two lexical representations: both its relevant English meaning (referring to a dessert made of pastry) and the irrelevant Polish one (since in Polish *pies* means *dog*). It remains unclear what processes underlie the ultimate selection of the appropriate language in bilingual comprehension, and more specifically, what processes underlie the selection of the appropriate meaning of an interlingual homograph. The Inhibitory Control Model (ICM) proposed by Green [5–7] suggested that the initial conflict between two languages, both in production and in comprehension, is resolved by a mechanism of active inhibition. Multiple studies have tested the model in the domain of language production (see [8] for a review of studies related to language production), but by far fewer in the domain of language comprehension [9–12].

Also, the most recent version of the well-known model of bilingual word recognition, Bilingual Interactive Activation Plus model (BIA+), assumes that interlingual homographs are represented by two (possibly partially overlapping) competing representations which interfere with each other on an orthographical level [13–14]. The different readings of a homograph are activated nonselectively and cannot be suppressed by local language context, so some control processes are needed for adequate lexical selection and task-related actions. However, the BIA+ model does not provide clear predictions describing how cross-language activation interference can be resolved. Therefore, in the present paper we focus on Green’s ICM as a theoretical model which has been previously successfully used to explain phenomena observed in bilingual production.

The main goal of the present paper is to explore whether the inhibitory mechanism may contribute to resolution of cross-language activation in bilingual comprehension and to investigate the possible influence of L2 proficiency on these processes. In what follows, we briefly consider available evidence on control processes in bilingual language comprehension and discuss the possible relation between these processes and L2 proficiency.

### Language non-selectivity, interference and mechanisms of control

As mentioned above, although initial co-activation of both of a bilingual's languages in comprehension has been documented across many studies and paradigms (both for words presented in isolation [15–17]; and for words presented in sentence context [18–22]), it is not clear how enduring this co-activation is, what its consequences for later stages of language processing are, and, critically, what mechanisms help to attenuate the initial activation of the irrelevant language. Going back to our initial example, for a Polish-English speaker, in order to correctly understand the utterance "Yesterday, she baked two pies," the initial activation of the non-target meaning of a homograph *pies* needs to somehow be attenuated in order to allow for the selection of the proper meaning. Although interlingual homographs are frequently used in research on bilingual comprehension and the plethora of data collected so far suggests that all the possible homograph’s meanings get activated regardless of the language context they are embedded into [2, 16, 23–24], it is unclear what mechanism resolves co-activation.

To assess the possible involvement of inhibition in resolving homograph interference in bilingual language comprehension, Macizo, Bajo and Martin (2010) contrived a task designed within the negative priming paradigm: they asked Spanish (L1)–English (L2) bilinguals to decide whether pairs of English words were semantically related to each other or not. In order to maintain the monolingual (L2 English) task context, all stimuli and instructions were presented in English and participants were not informed about the presence of interlingual homographs in the task. The task consisted of two-pair blocks. Table 1 illustrates the design and conditions of the task. To facilitate later presentation of the Polish-English task which was used in the current study, the table presents examples of both the original Spanish-English version of the task as well as the examples of stimuli from Polish-English adaptation of the task.

**Table 1:** Example of the structure of one experimental block.

***Pair 1***	**Homograph**		**NoHomograph**	
	Sp-Eng: toe—pie		Sp-Eng: log—pie	
	Pl-Eng: cat—pies		Pl-Eng: cat—art	
***Pair 2***	**Translation**	**NoTranslation**	**Translation**	**NoTranslation**
	Sp-Eng: hand—foot	Sp-Eng: hand—finger	Sp-Eng: hand—foot	Sp-Eng: hand—finger
	Pl-Eng: collar—dog	Pl-Eng: collar—neck	Pl-Eng: collar—dog	Pl-Eng: collar—neck

Each block consists of two pairs of words. The names of conditions are bolded. Each cell shows examples for Spanish-English stimuli (Sp-Eng) used in the original study by Macizo et al. (2010), and for Polish-English (Pl-Eng) stimuli used in the present study.

In *Pair 1*, two unrelated English words were presented. In the critical condition, a Spanish-English homograph (e.g., a word *pie*, meaning *foot* in Spanish) was presented along with a second word that was unrelated to the English meaning of the homograph. The second word in *Pair 1* was chosen to be associated with the non-target Spanish meaning of the homograph (e.g. *toe*). Therefore, the participant’s correct response in the semantic judgment task was "no", although the incorrect response "yes" was misleadingly primed by the Spanish reading of the homograph. In other words, the participant had to cope with a conflict raised by co-activation of two homograph’s meanings and ignore the Spanish meaning, which was irrelevant for the English context of the task. In the control condition the homographs were replaced with non-homographic English words (e.g. *log*). Macizo et al. (2010) reported that participants needed significantly more time to respond to pairs including homographs, and they produced over three times as many errors than in pairs consisting of only English words. The authors interpreted these between-condition differences as an index of co-activation and interference between both languages, and as evidence of language nonselective access in bilingual language processing. In *Pair* 2, two semantically related words were presented. In the critical condition, an English translation of the Spanish homograph meaning (e.g. *foot* as a translation of Spanish meaning of *pie*) was presented along with a related English word (e.g. *hand*). This condition was again compared against a control condition in which the translation was replaced with another English word related to the second word in *Pair 2* (e.g. *finger*). Conditions in *Pair 1* and *Pair 2* were fully crossed. Namely, half of the trials of *Pair 2* had a translation word, and half of the trials had a control word, for both the homograph and control conditions of *Pair 1*. Moreover, both *Pair 2* conditions were equally distributed across *Pair 1* conditions, such that both translation and control words in *Pair 2* were in half presented after the homograph condition and in half—after the control condition from *Pair 1*. The critical comparison involved response latencies and accuracy in the translation vs. control condition in *Pair 2* that were preceded by the homograph condition in *Pair 1*. As predicted, in the trials following the homograph condition in *Pair 1*, participants were slower and less accurate to respond to pairs containing homographs' translations than to the control pairs. In contrast, in trials following the control condition in *Pair 1*, no differences were observed between conditions in *Pair 2*. The authors argued that the observed interaction resulted from inhibition of the homograph's non-target meaning in *Pair 1*, which had to be reactivated [11] in *Pair 2*, so *Pair 2* provides an index of inhibitory control over competing meanings of the homographs. This pattern of results has been successfully replicated by authors in follow-up studies [12, 25].

Other recent studies have also reported the involvement of inhibition in bilingual language comprehension, although more indirectly. For example, Blumenfeld and Marian (2013) explored the role of inhibition in native spoken language comprehension, by comparing bilinguals and monolinguals in their ability to resolve phonological competition within a native language [26]. They found that bilinguals who were more efficient in suppressing interference from a language competitor were also more efficient in suppressing interference from irrelevant information in a nonlinguistic inhibitory task (Stroop); interestingly, the correlation was found only in bilinguals, and not in monolinguals. Also, Mercier, Pivneva and Titone (2013) reported that effective inhibitory control appears to help bilinguals overcome cross-language activation during spoken word comprehension, which suggests that the domain-general mechanism of inhibition plays a role in bilingual spoken word processing [13]. Similarly, Pivneva, Mercier and Titone (2014) demonstrated that individual abilities in executive control modulate cross-language activation during L2 reading [14]. The results of their recent study show that when bilinguals process homographs embedded in L2 sentences, individuals with more efficient executive control (measured with a battery of nonverbal inhibitory control tasks) experience reduced interference from irrelevant L1 interlingual homographs' meanings. Taken together, these studies provide preliminary evidence for the important role of executive control (and especially inhibition) in the resolution of cross-language activation in bilingual comprehension.

### Impact of L2 proficiency on interference between L1 and L2 and inhibitory control in bilingual comprehension

According to the Inhibitory Control Model [7] the amount of inhibition applied to resolve interference in language comprehension should be proportional to the level of activation of a given language [5, 7]. In other words, the higher the basic level of the non-target language activation, the more inhibition is required to suppress it. It thus follows that the strength of inhibition should be a function of L2 proficiency. When a bilingual uses her weaker L2, candidates from a more dominant L1 are strongly activated and compete with the target language candidates. The ICM proposes that the nontarget language needs to be inhibited. Moreover, the more strongly a particular representation has been inhibited, the longer time it requires to overcome inhibition and recover its normal activation level when the representation needs to be accessed again.

Previous research on language comprehension has not explicitly addressed the relation between L2 proficiency and the cognitive mechanisms underlying the resolution of co-activation between languages. In the studies that directly tested the involvement of inhibition in the resolution of homograph interference using the same semantic relatedness task as in the present study [11, 12, 25], participants’ linguistic profile was rather homogenous, especially in terms of L2 proficiency: they were late Spanish-English bilinguals, dominant in Spanish but highly proficient in English. Moreover, given the small number of participants in all the studies, the authors did not explore the potential influence of L2 proficiency differences.

### The present study

In the current study, we employed the paradigm described above—the semantic relatedness judgment task with interlingual homographs—and adapted it to a new set of languages: Polish (L1) and English (L2). Our first aim was to replicate the interference and inhibition effects observed in the original study with Spanish-English speakers, while the second aim was to test whether the inhibition effect depends on the participants' L2 proficiency.

The logic and the critical manipulations in the task used in the present study were identical to those of the original paradigm employed by Macizo et al. (2010) and Martin (2011). However, in order to minimize possible strategies that participants might use while responding to word pairs grouped by experimental blocks (i.e. *Pair 1* chunked with *Pair 2*), we modified the procedure so that the word pairs were grouped in a less intuitive way than in the original procedure. The original design seems to have both advantages and shortcomings. On the one hand, word pairs are grouped into clearly distinguishable two-word-pair chunks corresponding to the experimental blocks (e.g. a word pair containing a homograph chunked with a word pair containing its translation equivalent), so that activation (and possible inhibition) of words in *Pair 1* have more immediate carry-on effects on the words presented in *Pair 2*. On the other hand, this design might have enabled participants to see systematic relations between the words in *Pair 1* and the words in *Pair 2*. To minimize such strategic influences, we modified the timeline of the procedure, to make the grouping of stimuli into the blocks less salient. The details of the procedure modification will be presented in the Method section.

Based on the previous findings, we expected to observe a homograph interference effect in the Homograph condition in *Pair 1*; (i.e. co-activation of both homograph’s meanings and competition between them). The interference could stem from juxtaposition of the homograph with a word related to homograph’s L1 meaning, which causes a semantic conflict and enhances the competition between two homograph’s meanings. According to ICM, the interference between the homograph’s two meanings is resolved by the mechanism of inhibition, whose consequences should also be observed in *Pair 2*. Moreover, we predict that the magnitude of the interference effect in *Pair 1* will be negatively related to L2 proficiency: highly proficient bilinguals should experience less interference from a contextually irrelevant language (L1 or L2). The hypothesis is based on the idea that participants with low L2 proficiency have stronger residual activation of L1, relative to L2, which results in automatic activation of L1 meanings of homographs, even when the task is performed purely in L2 context [2, 3, 7, 10, 12, 18]. Therefore, at low levels of L2 proficiency, the contextually irrelevant L1 representations should strongly compete for selection with the task-relevant L2 representations. At higher levels of L2 proficiency, the initial strength of the task-relevant L2 meaning of the homograph should be higher, and the resulting interference easier to overcome [9].

In *Pair* 2, we expected to observe slower responses in the Translation condition following the Homograph condition in *Pair 1*, relative to the Translation condition following the NoHomograph condition in *Pair 1*. The slowdown should result from the difficulty of reactivating the L1 meaning of homograph that was inhibited in *Pair 1*. Based on the ICM [7], we hypothesize that the strength of inhibition (which consequences would be observed in *Pair 2)* is proportional to the strength of interference elicited in *Pair 1*: the stronger the interference from the irrelevant language, the more inhibition is needed to suppress it. Therefore, highly proficient bilinguals should not only experience less interference, but they should also engage less inhibition to suppress the irrelevant meaning of the homograph. Moreover, a similar effect (smaller homograph-related costs in the higher L2 proficiency group) could result from yet another source. As high L2 proficiency (or bilingualism) has been found to be related to more efficient nonlinguistic inhibitory control [27–29], participants with higher L2 proficiency might be able to ignore the irrelevant activation more effectively, i.e. using smaller amount of inhibition Specific predictions related to the task will be laid out after detailed presentation of the task in the Method section.

## Method

### Participants

The present experiment met the recommendations and requirements of the Ethics Committee in the Institute of Psychology concerning experimental studies with human subjects. Since our participants were underage, written informed consent for participation was given both by the participants and by their parents. Sixty nine first-year students from three secondary schools in Krakow volunteered to participate in the study (49 women, 20 men). Sixty three of them were right-handed and six were left-handed. Participants' mean age was 16.9 years (SD = 0.5). After completing the experimental session all participants received small gifts and were included in a prize lottery. All participants were unbalanced Polish–English bilinguals; they provided information about their language proficiency, learning history and their extent of language use by filling out a language background questionnaire based on Li, Sepanski, and Zhao’s (2006) questionnaire [30]. On average, they first started learning English in primary school at the age of 5.7 (SD = 2.2). However, for most participants, intensive contact with the L2 English began only at the age of 13. They also reported using mostly L1 during the day, since they were living in an L1 environment and used L2 only occasionally, mostly during English lessons at school. The participants considered their proficiency in L2 significantly lower than in L1 (for detailed self-assessment data see Table 2). To obtain an objective L2 proficiency measure, we also employed the Lexical Test for Advanced Learners of English, LexTALE [31]. The average LexTALE score in the tested group was 73% (SD = 10%) indicating that participants were moderately proficient in their L2.

**Table 2:** Means and standard deviations (in parentheses) of participants' proficiency and daily use of L1 and L2.

	L1	L2
**Self-rated proficiency**^a^	6.92 (0.08)	5.25 (0.15)
**a) Listening**	6.99 (0.11)	5.33 (0.98)
**a) Speaking**	6.93 (0.24)	5.09 (1.13)
**b) Reading**	6.96 (0.19)	5.44 (0.74)
**c) Writing**	6.79 (0.47)	5.15 (0.94)
**Percentage of daily use**^b^	79.55 (17.55)	16.43 (14.28)
**Semantic verbal fluency task (number of words)**^c^	22.14 (4.48)	15.48 (5.47)
**Phonological verbal fluency task (number of words)**^c^	11.58 (3.77)	10.71 (3.26)
**LexTALE (mean accuracy in %)**^d^	-	72.86 (10.49)

T-tests revealed that all ratings differed significantly between L1 and L2, *p*s < .05.

^a^ Self-ratings from a questionnaire with 1–7 scale, where 1 = no knowledge of given language, and 7 = native-like proficiency in given language.

^b^ Participants were asked to assess the percentage of daily use for every language, which would sum up to 100%. Some of the participants reported some knowledge and use of L3.

^c^ Number of words belonging to a given category (in semantic version) or beginning with a given letter (in phonological version) generated within one minute.

^d^ Aggregated percentage of correct answers for words and nonwords in lexical decision task; see [31].

To allow testing the hypotheses regarding the role of L2 proficiency in performing the semantic relatedness task, participants were post-hoc assigned to low and high L2 proficiency groups based on the median score in the LexTALE task (72%). Thirty six individuals below median were assigned to the low proficiency group (M = 66%, SD = 5%) and 33 who scored above were assigned to the high proficiency group (M = 80%, SD = 5%). The difference in LexTALE score between the groups was significant: *t*(67) = -11.69, p < .001.

### Semantic relatedness judgment task with interlingual homographs

#### Design

We developed an English-Polish version of the task that was originally designed by Macizo et al. (2010). In this task, participants were asked to assess whether words presented in a given pair were semantically related to each other or not. The words were displayed on a computer screen and participants indicated the yes/no responses by pressing one of the two designated keys on the keyboard. The entire task (including instructions) was conducted in participants’ L2 (English). It consisted of forty blocks of experimental items and twenty blocks of filler items; each block included two corresponding pairs of English words. *Pair 1* of each experimental block consisted of words unrelated to each other (i.e. the expected participant’s response was "no"). It belonged either to a Homograph condition or a NoHomograph condition. In the Homograph condition, a Polish-English homograph (e.g. *pies*, meaning dog in Polish) was paired with a word which was unrelated to the English meaning of the homograph, but related to the Polish one (e.g. *cat-pies)*. The NoHomograph condition served as a control condition, and so the homograph was replaced with a non-homograph control word that was semantically unrelated to the other word in *Pair 1* (e.g. *cat-art*; see Table 1 in the Introduction for a complete list of the experimental conditions and examples of stimuli). *Pair 2* in each block consisted of words semantically related to each other (i.e. the participant’s expected response was "yes"). It belonged to either a Translation condition or a NoTranslation condition. In the Translation condition, *Pair 2* consisted of an English translation of the Polish homograph meaning that was used *Pair 1* (e.g. *dog*), and a related English word (e.g. *collar-dog)*. The NoTranslation condition was designed as a control condition: the translation word was replaced with a control word which was semantically unrelated to the English meaning of the homograph from *Pair 1*, but was semantically related to the other word in *Pair 2* (e.g. *collar-neck)*.

Four versions of the task were created to counterbalance the stimuli material across conditions; no word appeared more than once in any version of the task. Each version consisted of 40 experimental blocks: 10 blocks in the NoHomograph-NoTranslation condition, 10 blocks in the NoHomograph-Translation condition, 10 blocks in the Homograph-NoTranslation condition, and 10 blocks in the Homograph-Translation condition (see Table 1). Blocks and conditions were fully counterbalanced across versions, such that no block appeared more than once per an experimental version, and each block occurred in all four conditions across versions. In each version of the task, every participant was exposed to each type of block ten times; each pair of words was presented once for each participant. In addition to the experimental blocks, 20 filler blocks were added to each list, identical throughout all four versions of the task. The filler blocks did not contain any homographs or words semantically related to any meaning of the homographs. Each filler block consisted of one pair of semantically related words (e.g. *sweet-candy*) and a following pair of unrelated words (e.g. *twice-sugar*). The second word *Pair 2* was always related to the words building *Pair 1* (e.g. *sugar* related to *sweet* and *candy*) so that the between-pair semantic correspondence was preserved, similarly to the experimental blocks. As a result, the filler blocks required “yes”-“no” responses, balancing the “no”-“yes” response order required in the experimental blocks. Presentation order of all blocks (both experimental and filler) was randomized.

#### Materials

Forty Polish–English homographs were used in the task. All homographs were legal words in both Polish and English. The logarithmized lexical frequency of the English meanings of the homographs (2.75, SD = 0.65), taken from SUBTLEX-US [32] was matched with the frequency of their Polish meanings (2.91, SD = 0.96), taken from a Polish version of SUBTLEX [33]. A t-test comparison showed no differences between frequencies of English meanings and Polish meanings of the homographs, *t* (39) = 0.99, *p* = .328. The homographs and their control words (see conditions’ description above) were matched with respect to mean number of letters and mean frequency, see Table 3. Similarly, lexical characteristics of translations and their control words were matched, see Table 3. Lexical characteristics of the stimuli were controlled using SUBTLEX-US [32]. In addition, stimuli were matched on two semantic characteristics: 1) concreteness (indicating how imaginable the designate of the given word is) and 2) meaningfulness (indicating how many other words are associated with the given word). In order to obtain these measures, an online pre-test was conducted in which participants were asked to estimate the concreteness (with a 1–5 scale, where: 1 = the word evokes an image immediately; 5 = the word evokes an image only after a long delay or not at all) and the meaningfulness (with a 1–7 scale, where: 1 –no associated word; 7 –many associated words) of all words yielding experimental blocks. 12 unbalanced Polish-English bilinguals with a good command of English (L2) completed the pre-test. A series of t tests showed that homographs and their control words, as well as homographs' translations and their control words, did not differ in the lexical and semantic characteristics (see Table 3 for results). The complete list of critical stimuli used in the task may be found in S1 Appendix and the list of words used in the filler blocks may be found in S2 Appendix.

**Table 3:** Means, standard deviations (in parentheses) and t-test for lexical and semantic characteristics of stimuli across conditions in the *Pair 1* and *Pair 2*.

*Pair 1*^a^			
	homograph	control	t—tests^c^
Mean number of letters	4.13 (0.72)	4.38 (0.93)	-1.43
Mean log frequency	2.75 (0.65)^d^	2.55 (0.5)	0.7
Mean concreteness	2.69 (1.03)	2.35 (0.94)	-2.72
Mean meaningfulness	3.36 (0.94)	3.32 (1.14)	-2.21
*Pair 2*^b^			
	translation	control	t—test
Mean number of letters	5.3 (1.71)	5.4 (1.13)	-0.31
Mean log frequency	2.46 (0.83)	2.80 (0.53)	-1.85
Mean concreteness	2.40 (0.98)	2.13 (0.96)	-0.33
Mean meaningfulness	3.35 (1.16)	3.50 (1.31)	1.32

^a^ The values characterize the critical word in *Pair 1*: the homograph word in the Homograph condition or a control word replacing the homograph in the NoHomograph condition.

^b^ The values characterize the critical word in *Pair 2*: the translation word in the Translation condition or a control word replacing the translation in the NoTranslation condition.

^c^ All ps > .05.

^d^ Only for English meanings of the homographs.

We conducted a pilot study to verify that the task measures the cross-language interference and inhibition of an irrelevant language only due to the presence of words ambiguous between languages and not by any other characteristics of the stimuli. A group of unbalanced Spanish-English bilinguals from the University of Granada (Spain) without any knowledge of Polish participated in the study. For reaction times, no significant differences were found either between the Homograph condition (1076 ms, SE = 53 ms) and the NoHomograph condition (1066 ms, SE = 52 ms) in the first pair (F < 1) or between the Translation condition (894 ms, SE = 41 ms) and the NoTranslation condition (907 ms, SE = 47 ms), F < 1. For error rates, we also did not observe any difference in Pair 1 between percentage of errors committed in the Homograph condition (26.59%, SE = 2.01%) and in the NoHomograph condition (17.95%, SE = 2.78%), F (1, 38) = 2.64, p > .05. In the second pair, participants were less accurate in the Translation condition (error rate 46.36%, SE = 2.61%) than in the NoTranslation condition (error rate 29.32%, SE = 3.13%), F (1, 78) = 5.6, p < .05. However, the difference was no longer present when eight items with exceptionally high error rates were excluded from the analysis. Overall, the results of the pilot study confirmed that the task would yield significant effects only for participants with a command of the Polish language.

#### Procedure

The experiment was run in DMDX [34]. Participants were tested individually in a soundproof room, seated approximately 60 cm from the screen. Stimuli were presented in lower-case white letters (Courier New font, size 10) on a black background. All the instructions were given in English; participants were also asked not to speak Polish, and in case that any questions were asked in Polish, they were answered in English in order to maintain the L2 monolingual context (following the recommendations of Grosjean, 2006; [35]). Participants were asked to decide whether presented pairs of words are semantically related to each other, and to respond as quickly and accurately as possible. Presence of Polish-English homographs was not mentioned. Responses were given with left and right shift keys, assigned to "yes" and "no" responses (counterbalanced across subjects). Before the proper task, a short training session was provided to familiarize participants with the experimental procedure. The training consisted of 12 blocks of exclusively English words that did not occur in the proper task (for the complete list of stimuli used in the training session see S3 Appendix). In line with modifications made by Martin (2011), to the original procedure by Macizo et al. (2010), the first and the second word of each pair was presented separately in order to adjust the procedure to the requirements of EEG measurement. Presentation of each word pair started with a fixation cross displayed centrally for 200 ms, followed by successive presentation of the first and second word of the word pair. The first word was presented for 500 ms, while the second word remained on the screen until reaction was given, or until a 2500 ms timeout passed. After a response to *Pair 1* was given (or after the timeout passed) the screen remained blank for 1500 ms, followed by the fixation mark preceding the second pair. A response to *Pair 2* was immediately followed by the fixation mark preceding *Pair 1* of a next block. The reported experiment was a part of the first stage of a three-stage longitudinal project. A large battery of experimental tasks and questionnaires was used in the project and both behavioral responses and EEG measures were recorded; the results from those tasks will be reported elsewhere.

### Predictions

Predictions were formulated separately for *Pair 1* and *Pair 2*. Following Macizo et al. (2010) we conceptualized the index derived in Pair 1 as a measure of interference and the index of Pair 2 as a measure of inhibition. Based on the previous results from the Spanish-English task, in *Pair 1* we expected to observe longer reaction times (RTs) for the word pairs including Polish-English homographs (the Homograph condition, e.g. *cat-pies*) than in control pairs without a homograph (the NoHomograph condition, e.g. *cat-art*) which could be interpreted as an index of cross-language interference. As mentioned at the end of Introduction, the interference should stem from the semantic conflict elicited by combining a homograph with a word related to its L1 meaning. To provide a correct response (i.e. to respond that words *cat* and *pies* are not related to each other), a participant has to resolve the interference. If resolution of the interference involves inhibition of the irrelevant homograph’s meaning, we should be able to observe the consequences of this inhibition in performance in Pair 2.

Specifically, *Pair 2* should lead to longer reaction times in the Homograph-Translation condition, i.e. in pairs including a translation of a Polish meaning of a homograph presented in *Pair 1* (e.g. *collar-dog*, following the presentation of *cat-pies*), relative to the other three conditions. We expected such pattern of results because the Homograph-Translation condition requires reactivation of a previously inhibited concept corresponding to the irrelevant Polish meaning of the homograph (e.g. the concept of a dog, corresponding to the Polish meaning of the homograph *pies* in *Pair 1*). As such, the delay observed in the Homograph-Translation condition in *Pair 2* (in comparison with the Homograph-NoTranslation condition) should be interpreted as an index of the inhibition that took place in *Pair 1*.

Additionally, we expected to observe an interaction between task performance and L2 proficiency level. Based on the IC model [7], we assumed that highly proficient bilinguals would experience less interference from the contextually irrelevant language, and whenever interference occurs, they would be more efficient in inhibiting the irrelevant language, relative to less proficient bilinguals. In consequence, we expected that in *Pair 1*, the low L2 proficiency group would reveal a stronger conflict between the two meanings of interlingual homographs (manifested as a larger difference in response between the Homograph and the NoHomograph condition). Moreover, if low L2 proficiency participants more strongly activated the irrelevant meaning of the homograph, they would need to apply stronger inhibition to overcome the resulting interference. Consequently, when the previously-inhibited meaning was presented in *Pair 2* (as the translation of the irrelevant homographs' meaning), low l2 proficiency participants should find it more difficult to reactivate the inhibited meaning, relative to highly proficient participants. This should manifest in longer response latencies in the Homograph-Translation condition as compared to the Homograph-NoTranslation condition.

## Results

Separate analyses were conducted for *Pair 1* and *Pair 2* of the semantic relatedness task with participants (F_1_) and items (F_2_) as the random variables. In RTs analysis, for each pair, subject and condition a median RT for correct trials was calculated. For accuracy, subject and condition mean error rates were calculated separately for *Pair 1* and *Pair 2*. In addition, we removed 10 items (from *Pair 2* only) from accuracy and RTs analyses as they had exceptionally high error rate (over 75%), indicating that the majority of participants did not know the English words building these pairs. Results from *Pair 1* were analyzed with a 2 x 2 ANOVA, with the *Pair 1* Condition (Homograph vs. NoHomograph) as a within-subject variable, and Proficiency (high L2 proficiency vs. low L2 proficiency) as a between-subject variable. For *Pair 2*, we conducted a 2 x 2 x 2 ANOVA, with *Pair 2* Condition (Translation vs. NoTranslation), and *Pair 1* Condition (Homograph vs. NoHomograph) as within-subject variables and Proficiency (high vs. low L2 proficiency) as a between-subject variable. All data from the experiment may be found in S1 Database.

### Pair 1

**Fig 1:**
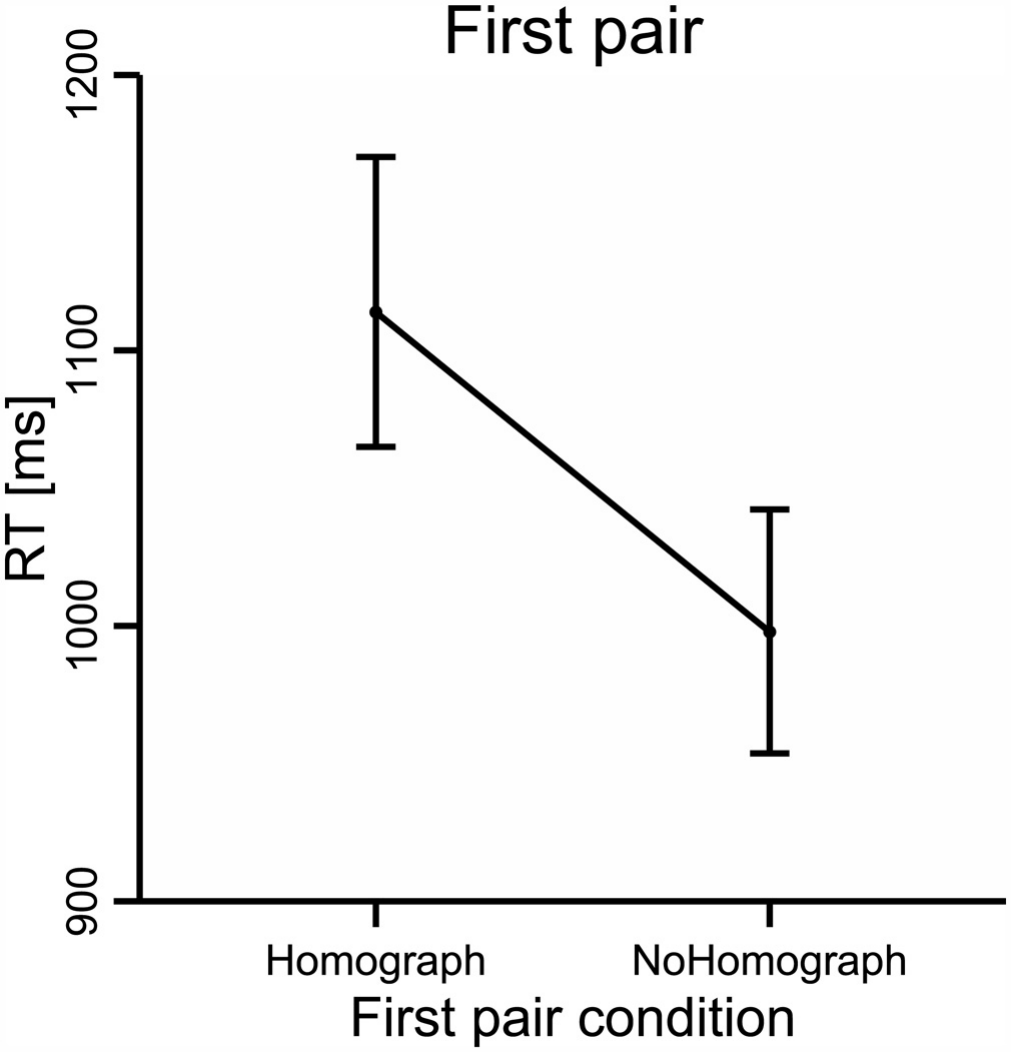
RTs in *Pair 1*.

The analysis of RTs revealed a main effect of *Pair 1* Condition: F_1_ (1, 68) = 38.38, p < .001, partial η2 = .36, F_2_ (1, 78) = 13.03, p < .001, partial η2 = .14; participants took significantly longer to respond in the Homograph condition (1114 ms, SD = 225 ms) than in the NoHomograph condition (998 ms, SD = 186 ms; see Fig 1). In addition, there was a main effect of Proficiency: F_1_(1, 68) = 12.79, p < .01, partial η2 = .16, F_2_ (1, 78) = 71.91, p < .001, partial η2 = .48; highly proficient participants responded faster (976 ms, SD = 178 ms) than less proficient participants (1129 ms, SD = 176 ms). However, no interaction between Proficiency and *Pair 1* Condition was observed (F_1_ and F_2_ < 1).

The accuracy analysis brought a similar pattern of effects. There was a main effect of *Pair 1* Condition: F_1_ (1, 68) = 131.42, p < .001, partial η2 = .67, F_2_ (1, 78) = 40.15, p < .001, partial η2 = .34, caused by more errors the Homograph condition (error rate 31.19%, SD = 15.14%) than the NoHomograph condition (error rate 12.09%, SD = 12.15%). The main effect of Proficiency was also significant: F_1_(1, 68) = 8.38, p < .01, partial η2 = .12, F_2_ (1, 78) = 27.21, p < .001, partial η2 = .26; highly proficient participants committed fewer errors (error rate 17.5%, SD = 10.6%) than less proficient participants (error rate 25.44%, SD = 11.8%). Again, no interaction between Proficiency and *Pair 1* Condition was observed (F_1_ and F_2_ < 1).

### Pair 2

**Fig 2:**
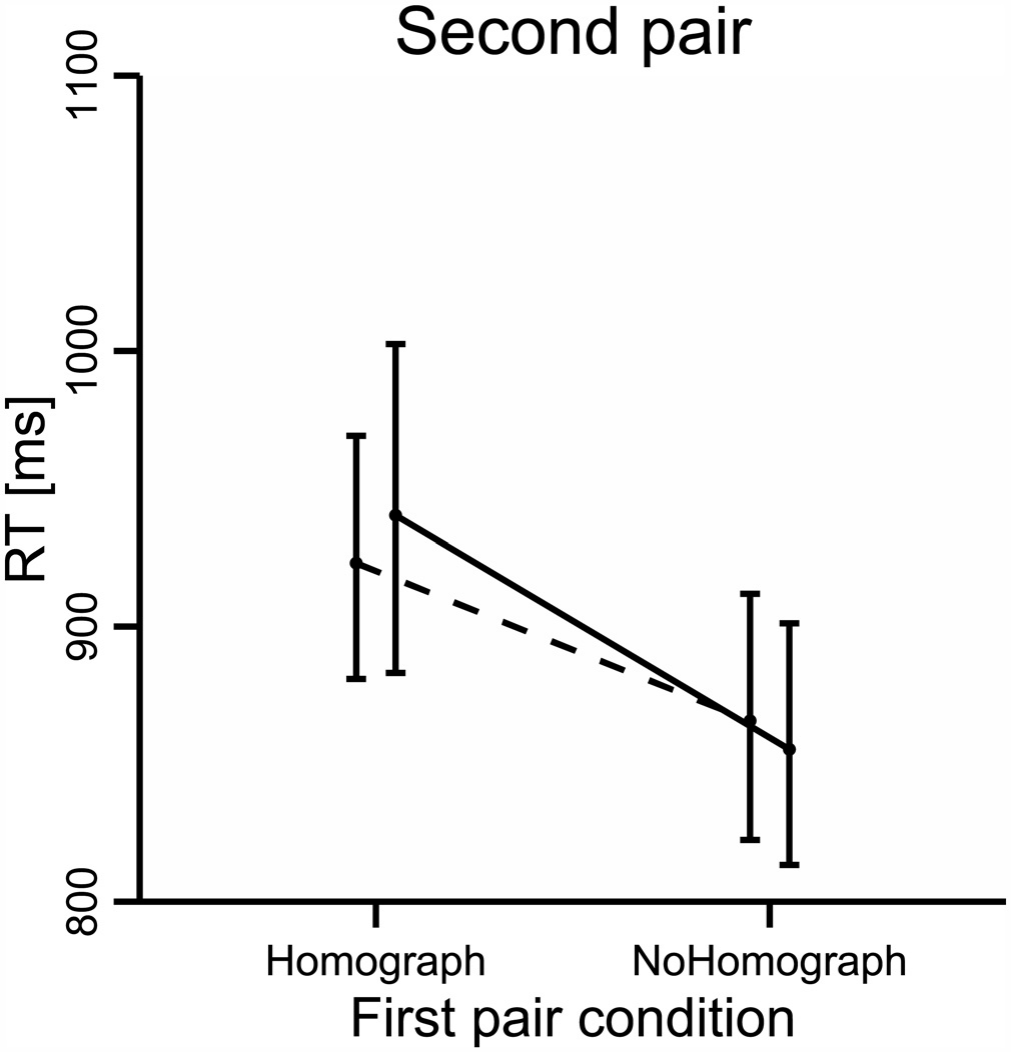
RTs in *Pair 2*.

The analysis of RTs revealed a main effect of *Pair 1* Condition: F_1_(1, 68) = 20.7, p < .001, partial η2 = .23, F_2_ (1, 78) = 10.3, p < .01, partial η2 = .13; participants took longer to respond when *Pair 2* was preceded by *Pair 1* in the Homograph condition (931 ms, SD = 190 ms) than when it was preceded by *Pair 1* in the NoHomograph condition (861 ms, SD = 165 ms). However, contrary to our predictions, there was neither a significant main effect of *Pair 2* Condition (all Fs < 1), nor an interaction between *Pair 1* Condition and *Pair 2* Condition (all Fs < 1; see Fig 2). In the analysis by subject, participants with high and low proficiency responded equally fast in *Pair 2* (F_1_ < 1), while in the analysis by item, participants showed a main effect of Proficiency: F_2_ (1, 78) = 27.53, p < .001, partial η2 = .29. There was no interaction between Proficiency and any of the task conditions.

In the accuracy analysis, no main effect of *Pair 1* Condition was observed (F_1_ and F_2_ < 1). However, there was a significant main effect of *Pair 2* Condition: F_1_ (1, 68) = 35.84, p < .001, partial η2 = .35, F_2_ (1, 78) = 3.96, p = .05, partial η2 = .06; participants obtained higher error rates in the Translation condition (31.51%, SD = 14.94%) than in the NoTranslation condition (21.64%, SD = 13.17%). There was no interaction between *Pair 1* Condition and *Pair 2* Condition: F_1_(1, 68) = 1.1, p = .1, partial η2 = .02, F_2_ (1, 78) = 3.12, p > .05, partial η2 = .04. We observed a significant main effect of Proficiency: F_1_ (1, 68) = 30.57, p < .001, partial η2 = .31, F_2_ (1, 78) = 36.02, p < .001, partial η2 = .35; participants with lower L2 proficiency committed more errors than participants with higher L2 proficiency (33.13%, SD = 10.51% versus 19.43%, SD = 10.01%, respectively). There was no interaction between Proficiency and any of the task conditions (all Fs < 1).

## Discussion

The present study explored mechanisms that allow bilinguals to select between two languages during language comprehension. For this purpose, a group of late unbalanced Polish-English bilinguals completed a semantic relatedness task employing Polish-English interlingual homographs. The task consisted of series of blocks composed of two word pairs; for each word pair participants were asked to judge their semantic relatedness (see Table 1 in the Introduction). *Pair 1* consisted of unrelated English words, and thus the expected answer was "no.” In the critical condition (the Homograph condition), one word in *Pair 1* was a Polish-English homograph (e.g. *pies*, meaning dog in Polish), while the second word in *Pair 1* was unrelated to the English meaning of the homograph, but related to its Polish meaning (yielding e.g. *pies-cat*). The Homograph condition was compared with a control condition (the NoHomograph condition) in which the homograph was substituted with a matched non-homograph (yielding e.g. *art-cat*). In line with our predictions, participants were slower when responding to pairs including homographs. This indicates that, at least initially, both meanings of homographs were non-selectively activated, even though the task was conducted exclusively in the L2 context. These results are consistent with between-language interference effects observed earlier in the experiments using the Spanish-English version of the task [11, 25] and provide further evidence for non-selective language activation in language comprehension in bilinguals [2]. As such, the results seem consistent with both ICM and BIA+ models, which both predict interference between two alternative readings of the homograph and posit that it is not possible to prevent the activation of the irrelevant homograph’s meaning, even when it is required by a given task and would undoubtedly improve task performance [9, 10, 36].

*Pair 2* of each experimental block consisted of semantically related words and thus required a "yes" answer. In the Translation condition, the Polish meaning of the previously used homograph was translated into English (e.g, *dog*, being the translation of the Polish meaning of the homograph *pies*). The translation was paired with a semantically related word (yielding e.g. *dog-collar*). In the NoTranslation condition, the homograph's translation was substituted with a matched control word (yielding e.g. *collar*—*neck*). When *Pair 1* included a homograph and *Pair 2* included its translation (Homograph-Translation condition), then participants needed to reactivate the previously task-irrelevant meaning of the homograph. Thus, the comparison between the Homograph-Translation and the Homograph-NoTranslation condition provided an index of the effort needed to reactivate the previously irrelevant homograph meaning. Based on the results obtained by Macizo et al. (2010) and Martin (2011), we expected longer reaction times in the Homograph-Translation condition relative to the NoHomograph-Translation condition. We also expected that the Homograph-Translation condition would also be more difficult than the two conditions of *Pair 2* which did not include the translation of the homograph (i.e. the Homograph-NoTranslation and the NoHomograph-NoTranslation conditions).

Contrary to our expectations and to previous findings by Macizo et al. (2010) and by Martin (2011), both conditions in which the homograph occurred in *Pair 1* (the Homograph-Translation and the Homograph-NoTranslation conditions) led to slower reaction times, relative to the conditions in which *Pair 1* did not contain a homograph (the NoHomograph-Translation and the NoHomograph-NoTranslation; see Table 1 in the Introduction). As such, the results are incompatible with the interpretation that only the irrelevant meaning of the homograph gets inhibited. We believe that the unexpected pattern of results can be accounted for by a broader scope of inhibition that took place in Pair 1, and by a relatively low L2 proficiency of the participants in the current study. We will elaborate this hypothesis in the following section.

It should be also noted that, in addition to the reaction time results summarized above, in *Pair 2* we also observed a general decrease in accuracy in the Translation relative to the NoTranslation condition. This replicates the original Spanish-English studies that also revealed larger error rates in the same conditions of *Pair 2*. Although Macizo et al. (2010) did not elaborate on this finding, we hypothesize that words in the Translation condition were less familiar to the unbalanced participants tested in the current study, despite the fact that these words were matched in frequency. It should be noted, however, that the condition of *Pair 2* (Translation vs. NoTranslation) did not interact with the condition of *Pair 1* (Homograph vs. NoHomograph), indicating that accuracy *Pair 2* did not depend on the presence or absence of a homograph in *Pair 1*.

### The role of L2 proficiency

One of the goals of the current study was to explore how L2 proficiency modulates the selection between alternative meanings of homographs and how it affects the inhibition of their irrelevant meaning. At the outset of the study, we hypothesized that bilinguals with lower L2 proficiency would experience stronger interference from the task-irrelevant L1 meaning of homographs and would need to engage stronger inhibitory processes in order to overcome the interference. Hence, for participants with lower L2 proficiency, we expected to observe more interference in the Homograph condition in *Pair 1* and a stronger inhibitory effect in the Homograph-Translation condition in *Pair 2*, relative to participants with higher L2 proficiency. To verify these hypotheses, we derived an L2 proficiency variable based on median-split of LexTALE scores, and tested whether it modulates the magnitude of the experimental effects. The results indicate that L2 proficiency did not interact with critical manipulations in the task, neither in the first nor in the second pair. As indicated in the Method section, the data reported in the present paper comes from a study in which a large battery of tasks was used. Although the reported analyses are based on the LexTALE score as the L2 proficiency index, we also performed parallel analyses using other available measures of L2 proficiency, to see if any of them modulate the basic pattern of results. Two tasks were used for this purpose: a semantic verbal fluency task in English and a picture naming task in English. All three measures of L2 proficiency correlated with each other moderately, but significantly. None of the indices of L2 proficiency affected the magnitude of the interference and the inhibition effects. One could hypothesize that the variability in L2 proficiency was too small to affect performance in the semantic relatedness task. However, this explanation seems to be unlikely, as the two groups of participants significantly differed in their L2 proficiency measured with LexTALE [31]. Moreover, L2 proficiency did affect the overall performance in the task: relative to highly proficient group, participants in the low proficient group made more errors and were slower in the first pair (see the Results section for the report of the main effect of proficiency in *Pair 1*). Thus, the lack of impact of L2 proficiency on the interference and inhibition effects suggests that there is no relation between L2 proficiency and the strength with which the L1 and L2 meanings of a homograph are activated (and subsequently inhibited), at least in the range of L2 proficiency represented in our sample. It remains an open question whether the strength of interference and inhibition would differ if the two compared groups were even more heterogeneous in terms of their L2 proficiency.

Following a reviewer’s suggestion, we conducted post-hoc analyses, comparing participants from the highest and lowest quartile of L2 proficiency (based on their scores in LexTALE). The comparison, however, did not yield any significant results. Additionally, we performed analyses including measures of individual differences in working memory capacity, IQ and nonlinguistic cognitive control (measured with Eriksen Flanker Task), but no significant correlations between these measures and the main effects in Pair 1 and Pair 2 were observed.

We hypothesize, however, that L2 proficiency is crucial for a different aspect of the results. More specifically, we propose that differences in L2 proficiency could explain the differences between the results of the current study and those by Macizo et al. (2010) and Martin (2011). While the previous studies show inhibitory effects only when *Pair 2* contained a translation of the homograph presented in *Pair 1*, the data from the current study demonstrate a general inhibitory effect in the second pair following a homograph, which is not limited to the translation of the homograph. We speculate that the discrepancy in the outcomes of the study’s results from a differential scope of inhibition induced by homographs presented in *Pair 1* which varied across the studies. Since participants of our study were less proficient than the Spanish-English participants (as we show in more detail below), we propose that the difference in L2 proficiency across the studies may have impacted the scope of inhibition induced by *Pair 1*. In the following section, we elaborate on this proposal.

### The scope of inhibition

In the task employed in the current study participants were explicitly asked to assess semantic relations between English (L2) words. Although the L2 meaning of the homograph was never semantically related to the other word in *Pair 1*, the L1 meaning of the homograph always was. Thus, in the Homograph condition the two words might have been perceived as semantically related, and activated a semantic category jointly determined by them. To illustrate, the pair *cat-pies* may have been initially understood as *cat-dog*, since *pies* means "dog" in Polish and the two concepts jointly activated the category of domestic animals. This would not happen if only the L2 meaning of the homograph (*pies* meaning desserts made of pastry) was activated. It would also not be the case in the NoHomograph condition (*cat-art*), where the category of domestic animals is presumably activated to much smaller extent by the sole word *cat*, than by the concepts of dog and cat elicited jointly in the Homograph condition. This broad activation of a semantic category should be particularly pronounced for highly unbalanced bilinguals, who activate the L1 meaning of a homograph more strongly than its L2 meaning [23, 24, 37–39], such as participants in our study. As mentioned before, the Polish-English speakers who participated in the present study were less proficient in their L2 and less language-balanced than the Spanish-English participants of the study by Macizo et al. (2010). Since the experimental task required that participants determine whether the two words are related, the less proficient bilinguals could have initially perceived the two words of the first pair in the Homograph condition as related to each other. In order to give the correct (negative) response, participants had to inhibit the whole semantic category determined by the two words. In contrast to speakers with low L2 proficiency, those with high L2 proficiency activate the L1 meaning of the homograph less strongly. Thus, for speakers who are more proficient in L2 than those tested in the current study, the irrelevant L1 meaning of the homograph (e.g. “dog” as the Polish meaning of *pies*) and the other word in *Pair 1* (e.g. *cat*) might be less likely to co-activate a common semantic category and instead may activate just the target word (as observed by Macizo et al. 2010). However, in the group of participants with a relatively low L2 proficiency (as in the current study), activation of the irrelevant L2 meaning of the homograph might lead to co-activation of the whole semantic category. As such, participants with higher and lower L2 proficiency may differ in the scope of inhibition induced by *Pair 1* containing homographs: highly proficient bilinguals may inhibit only the irrelevant meaning of the homograph, whereas less proficient bilinguals may inhibit a whole semantic category related to the irrelevant meaning of the homograph and to the other word in *Pair 1*.

Variation in the scope of inhibition should have ramifications for the processing of *Pair 2*. By virtue of the task design (see Method section), the words in *Pair 2* were semantically related to the irrelevant meaning of the homograph, i.e. they belonged to the same semantic category. At the same time, it was the same category which was irrelevantly elicited (and subsequently inhibited) in *Pair 1* in the Homograph condition. Thus, after the Homograph condition, the response in *Pair 2* was hampered both in the Translation condition (*collar-dog*) and in the NoTranslation condition (*collar-neck*) because in both conditions the words of *Pair 2* were semantically related to the previously inhibited category (e.g. the category of domestic animals). In contrast, when *Pair 1* did not include any homograph, there was no need to overcome any inhibition because, as we explained earlier, in that case *Pair 1* did not lead to the activation and subsequent inhibition of homograph’s meaning or a category co-determined by a homograph. To sum up, the current study suggests that the scope of activation and inhibition in a semantic relatedness paradigm may be wider than expected, and this possibility should be considered both designing tasks within this paradigm and interpreting data gathered with them.

We assume that the variation in scope of activation and inhibition of semantic categories was elicited by a difference in proficiency between participants of our study and the participants of the study by Macizo et al. (2010) and Martin (2011). Participants in our study were teenage secondary school students, whereas the participants in both Spanish-English studies were university students from the English linguistics department [11, 12, 25]. Although a direct comparison between them is not possible, it seems very likely that the Spanish-English participants were more proficient in English and used their L2 more frequently and extensively, which made them more balanced bilinguals overall. A comparison of average reaction times and accuracy between the Polish-English and the Spanish-English bilinguals clearly shows an advantage of the Spanish-English group. In the first pair, where the procedure in the current study was identical with the procedure used with the Spanish-English bilinguals, Polish-English participants were on average 200 ms slower and about 10% less accurate than the Spanish-English participants in the study by Martin (2011), which confirms our intuition about differences in L2 proficiency between the groups. Within the present study we did not observe any variation in scope of inhibition depending on L2 proficiency of our participants, probably due to too-small variability in L2 proficiency in the tested group.

### Time course of inhibitory control in language comprehension—insights from a comparison with previous studies

As mentioned earlier, in the present study we modified the semantic judgment task procedure to minimize the chance that participants engage into any strategies while performing the task. In essence, we re-grouped the word pairs so that *Pair 2* of a previous block and *Pair 1* of the next block were grouped together. In doing so, we expected that participants will be less likely to notice the relation between *Pair 1* and *Pair 2* of a block, than in the original study by Macizo et al. (2010). Not noticing the relation would also prevent participants from developing any potential strategies related to task performance (e.g. generating a hypothesis about the upcoming translation of the homograph).

An additional consequence of this change was longer interval between the presentation of the homograph and the onset of the first word in *Pair 2* (the interval was 1000 ms longer than in the original procedure used by Macizo et al., 2010). Therefore, apart from preventing participants from developing strategies based on relatedness of the two pairs, the change also provided a harder test for the persistence of the inhibitory processes in comprehension. The results indicate that despite the longer interval between *Pair 1* and *Pair 2*, we still observed the interference effects in *Pair 2* following the Homograph condition, relative to *Pair 2* following the NoHomograph condition. Thus, it seems that the effect of inhibition elicited in *Pair 1* was quite long-lasting and endured over one second. This result is contrary to the results obtained by Martin et al. (2010), where the inhibitory effect occurred only when the between-trial interval lasted 500 ms, but not when the between-trial interval lasted 750 ms. A possible explanation of the more persistent inhibitory effects observed in the current study relates to the low L2 proficiency of participants in the current study: due to a big difference between resting activation levels of L1 and L2, the irrelevant L1 representations needed to be more strongly inhibited, and this strong inhibition lasted longer, than in the previous studies testing more proficient and balanced participants (Martin et al., 2010). However, at this point, such an interpretation is only a speculation and requires systematic testing. We are currently examining this issue in our laboratory.

## Conclusions

To sum up, the present study brings new evidence for co-activation and competition between languages in bilingual comprehension. The results demonstrate that even in an exclusively L2 context bilinguals activate both L1 and L2 meanings of interlingual homographs and that there is a robust interference between these two meanings. The data are consistent with the notion that inhibitory processes are required to resolve the interference and inhibit the irrelevant homograph meaning. Additionally, the present study extends the previous findings by demonstrating the flexibility of the inhibitory mechanisms: the inhibitory processes appear to impact not only specific lexical items but a broader semantic category activated by an exemplar used in the task.

## Supporting Information

S1 Appendix Critical stimuli. (PDF)

S2 Appendix Stimuli in the filler blocks. (PDF)

S3 Appendix Stimuli in the training session. (PDF)

S1 Database RTs & ERs in Polish-English homograph task. (TXT)

## References

[1] Schwartz AI, Van Hell JG. Bilingual visual word recognition in sentence context In: Adelman J, editor. *Visual Word Recognition*. Psychology Press; 2012.

[2] Van Assche E, Duyck W, Hartsuiker RJ. Bilingual word recognition in a sentence context. Front Psych. 2012; 3: 1–8. 10.3389/fpsyg.2012.00174 PMC3365650 22675314

[3] Dijkstra T, van Heuven WJB. The architecture of the bilingual word recognition system: From identification to decision. Biling Lang Cogn. 2002; 5(03).

[4] Schwartz AI, Kroll JF, Diaz M. Reading words in Spanish and English: Mapping orthography to phonology in two languages. Language and Cognitive Processes. 2007; 22, 106–129.

[5] Abutalebi J, Green DW. Bilingual language production: The neurocognition of language representation and control. J Neurolinguist. 2007; 20, 242–275.

[6] Abutalebi J, Green DW. Control mechanisms in bilingual language production: Neural evidence from language switching studies. Language and Cognitive Processes. 2008; 23, 557–582.

[7] Green DW. Mental control of the bilingual lexico-semantic system. Biling Lang Cogn. 1998; 1(2), 67–81.

[8] Kroll JF, Gollan TH. Speech planning in two languages: What bilinguals tell us about language production In: Ferreira V. M Goldrick M, Miozzo M, editors. The Oxford Handbook of Language Production (pp. 165–181). Oxford: Oxford University Press; 2014.

[9] Macizo P, Bajo T, Cruz Martín M. Inhibitory processes in bilingual language comprehension: Evidence from Spanish–English interlexical homographs. J Mem Lang. 2010; 63(2): 232–44.

[10] Martín MC, Macizo P, Bajo T. Time course of inhibitory processes in bilingual language processing. Br J Psychol. 2010; 101(4):679–93. 20184787 10.1348/000712609X480571

[11] Mercier J, Pivneva I, Titone D. Individual differences in inhibitory control relate to bilingual spoken word processing. Biling Lang Cogn. 2013; 17(01):89–117.

[12] Pivneva I, Mercier J, Titone D. Executive control modulates cross-language lexical activation during L2 reading: evidence from eye movements. J Exp Psychol Learn Mem Cogn. 2014; 40(3): 787–96. 10.1037/a0035583 24446754

[13] Dijkstra T, van Heuven WJB. The architecture of the bilingual word recognition system: From identification to decision. Biling Lang Cogn. 2002; 5, 175–197.

[14] Van Heuven WJB, Dijkstra T. Language comprehension in the bilingual brain: fMRI and ERP support for psycholinguistic models. Brain Res Rev. 2010; 64(1):104–22. 10.1016/j.brainresrev.2010.03.002 20227440

[15] Bijeljac-Babic R, Biardeau A, Grainger J. Masked orthographic priming in bilingual word recognition. Mem Cognit; 1997; 25, 447–457. 9259623 10.3758/bf03201121

[16] Dijkstra T, Van Jaarsveld H, Brinke S. Interlingual homograph recognition: Effects of task demands and language intermixing. Biling Lang Cogn. 1998; 1(1):51–66.

[17] Van Heuven WJB, Dijkstra T, Grainger J. Orthographic Neighborhood Effects in Bilingual Word Recognition. J Mem Lang; 1998; 39(3):458–83.

[18] Duyck W, Van Assche E, Drieghe D, Hartsuiker RJ. Visual word recognition by bilinguals in a sentence context: evidence for nons- elective lexical access. J. Exp. Psychol. Learn.Mem. Cogn. 2007; 33, 663–679. 17576146 10.1037/0278-7393.33.4.663

[19] Elston-Güttler KE, Gunter TC, Kotz S. Zooming into L2: global language context and adjustment affect processing of interlingual homographs in sentences. Brain Res Cogn. 2005; 25(1):57–70. 10.1016/j.cogbrainres.2005.04.007 15905078

[20] Schwartz AI, Kroll JF. Bilingual lexical activation in sentence context. J Mem Lang. 2006; 55(2):197–212.

[21] Van Assche E, Duyck W, Hartsuiker RJ, Diependaele K. Does bilingualism change native-language reading? Cognate effects in a sentence context. Psychological Science. 2009; 20, 923–927. 10.1111/j.1467-9280.2009.02389.x 19549082

[22] van Hell JG, Dijkstra T. Foreign language knowledge can influence native language performance in exclusively native contexts. Psychon Bull Rev. 2002; 9, 780–789. 12613683 10.3758/bf03196335

[23] Dijkstra T, Timmermans M, Schriefers H. On Being Blinded by Your Other Language: Effects of Task Demands on Interlingual Homograph Recognition. J Mem Lang. 2000; 42(4):445–64.

[24] Kerkhofs R, Dijkstra T, Chwilla DJ, de Bruijn ER. Testing a model for bilingual semantic priming with interlingual homographs: RT and N400 effects. Brain Res. 2006; 1068(1):170–83. 16375868 10.1016/j.brainres.2005.10.087

[25] Martin MC. Inhibitory control in bilingualism. Unpublished doctoral thesis. University of Granada; 2011.

[26] Blumenfeld HK, Marian V. Parallel language activation and cognitive control during spoken word recognition in bilinguals. J Cogn Psychol. 2013; 25(5):37–41. 10.1080/20445911.2013.812093 PMC3827904 24244842

[27] Colzato LS, Bajo MT, van den Wildenberg W, Paolieri D, Nieuwenhuis S, La Heij W, et al How does bilingualism improve executive control? A comparison of active and reactive inhibition mechanisms. J Exp Psychol Learn Mem Cogn. 2008; 34(2):302–12. 10.1037/0278-7393.34.2.302 18315407

[28] Khare V, Verma A, Kar B, Srinivasan N, Brysbaert M. Bilingualism and the increased attentional blink effect: evidence that the difference between bilinguals and monolinguals generalizes to different levels of second language proficiency. Psychol Res. 2013; 77(6):728–37. 10.1007/s00426-012-0466-4 23196431

[29] Tao L, Marzecová A, Taft M, Asanowicz D, Wodniecka Z. The efficiency of attentional networks in early and late bilinguals: the role of age of acquisition. Frontiers in Psychology. 2011; 2:123 doi: 0.3389/fpsyg.2011.00123 21713011 10.3389/fpsyg.2011.00123 PMC3114252

[30] Li P, Sepanski S, Zhao X. Language history questionnaires: A web-based interface for bilingual research. Behavioral Research Methods. 2006; 38, 202–210. 10.3758/bf03192770 16956095

[31] Lemhöfer K, Broersma M. Introducing LexTALE: a quick and valid Lexical Test for Advanced Learners of English. Behav Res Methods. 2011; 44(2):325–43. 10.3758/s13428-011-0146-0 PMC3356522 21898159

[32] Brysbaert M, New B. Moving beyond Kučera and Francis: A critical evaluation of current word frequency norms and the introduction of a new and improved word frequency measure for American English. Behavior research methods. 2009; 41(4), 977–990. 10.3758/BRM.41.4.977 19897807

[33] Mandera P, Keuleers E, Wodniecka Z, Brysbaert M. Subtlex-pl: subtitle-based word frequency estimates for Polish. Behavior Research Methods. 2014 (online publication). 10.3758/s13428-014-0489-4 24942246

[34] Forster KI, Forster JC. DMDX: A windows display program with millisecond accuracy. Behavior Research Methods, Instruments, &Computers. 2003; 35 (1), 116–124. 10.3758/bf03195503 12723786

[35] Grosjean F. Studying bilinguals: Methodological and conceptual issues In: Bhatia TK, Ritchie WC, editors. The Handbook of Bilingualism (pp. 32–63). Oxford: Blackwell Publishing; 2006.

[36] Lemhöfer K, Dijkstra T. Recognizing cognates and interlingual homographs: effects of code similarity in language-specific and generalized lexical decision. Memory & Cognition. 2004; 32(4), 533–50. 10.3758/BF03195845 15478748

[37] Elston-Güttler KE, Paulmann S, Kotz S a. Who’s in control? Proficiency and L1 influence on L2 processing. J Cogn Neurosci. 2005; 17(10):1593–610. 16269099 10.1162/089892905774597245

[38] Sunderman G, Kroll JF. First language activation during second language lexical processing: An investigation of lexical form, meaning, and grammatical class. Studies in Second Language Acquisition. 2006; 28, 387–422.

[39] Elston-Güttler KE, Friederici AD. Native and L2 processing of homonyms in sentential context. J Mem Lang. 2005; 52(2):256–83.

