# Supplementary material for: Interference and Inhibition in Bilingual Language Comprehension: Evidence from Polish-English Interlingual Homographs
Source: PLoS One. 2016 Mar 15;11(3):e0151430. doi: 10.1371/journal.pone.0151430 (PMC4792378; doi:10.1371/journal.pone.0151430)
Supplement: S1 Appendix — (PDF) [file pone.0151430.s001.pdf]

Block number	First pair			Second pair		
	First word in pair	Second word in pair		First word in pair	Second word in pair	
		homograph	control		translation	control
1	cat	pies	art	collar	dog	neck
2	grass	pole	glove	cows	field	bulls
3	foot	but	ant	leather	shoe	belt
4	wardrobe	mole	pencil	flame	moths	torches
5	horse	fury	pine	wheel	carts	fortune
6	new	stare	dry	young	old	kittens
7	head	nosy	float	throat	nose	tongue
8	guys	baby	trap	girls	women	boys
9	eye	brew	aid	forehead	eyebrow	wrinkle
10	water	wanna	lorry	tub	bath	wash
11	bang	race	gold	display	fireworks	fashion
12	insect	much	oily	buzz	flies	bee
13	candle	knot	tiger	lamp	wick	shade
14	king	car	bulb	Russia	tzar	vodka
15	canyon	jar	pea	valley	gorge	mountain
16	hair	rude	frog	ginger	redhead	spice
17	bird	paw	lawn	feather	peacock	swans
18	beard	cap	onion	milk	goat	shake
19	black	bury	whom	white	grey	snow
20	lonely	same	seal	together	alone	apart
21	skin	blade	scone	face	pale	mouth
22	ever	stale	peak	never	always	again
23	river	most	spoon	over	bridge	above
24	lift	windy	stew	stairs	elevators	climb
25	sick	chore	jugs	well	ill	badly
26	oven	piece	lion	heat	stoves	warmth
27	beer	lane	sand	spill	poured	liquid
28	school	sale	tin	door	rooms	handle

Block number	First pair			Second pair		
	First word in pair	Second word in pair		First word in pair	Second word in pair	
		homograph	control		translation	control
29	hole	jam	song	dark	pits	bright
30	morning	rosy	chain	wet	dew	rain
31	spirit	duchy	account	haunt	ghosts	castle
32	chair	stole	sign	cloth	table	coat
33	drunk	lump	wallet	whisky	alcoholic	brandy
34	food	post	mirror	eat	feast	meal
35	earth	grunt	frame	floor	ground	carpet
36	hare	chart	sword	hunt	greyhound	chase
37	skeleton	gnat	ruled	flesh	bone	muscle
38	wood	chat	corn	sheds	huts	garden
39	green	lip	slave	tree	limes	leaf
40	money	hazard	wolf	cards	gambling	playing
