# Supplementary material for: Interference and Inhibition in Bilingual Language Comprehension: Evidence from Polish-English Interlingual Homographs
Source: PLoS One. 2016 Mar 15;11(3):e0151430. doi: 10.1371/journal.pone.0151430 (PMC4792378; doi:10.1371/journal.pone.0151430)
Supplement: S2 Appendix — (PDF) [file pone.0151430.s002.pdf]

	First word in pair	Second word in pair
Block 1	mist	fog
	timber	cloud
Block 2	sweet	candy
	twice	sugar
Block 3	fish	sea
	log	wave
Block 4	print	book
	price	page
Block 5	pill	cure
	skip	doctor
Block 6	sky	sun
	tie	shine
Block 7	fruit	plum
	pony	apricot
Block 8	straw	meadow
	trace	flower
Block 9	toe	leg
	his	hand
Block 10	rail	train
	soul	ticket
Block 11	gate	fence
	poster	path
Block 12	soup	plate
	safe	fork
Block13	ring	silver
	leaflet	necklace
Block 14	scissors	trim
	sock	comb
Block 15	paint	brush
	towel	draw
Block 16	call	mobile
	mug	phone
Block 17	post	stamp
	brake	letter
Block 18	tower	castle
	tomato	king
Block 19	class	pupil
	brain	desk
Block 20	plant	root
	edge	bud
