# Supplementary material for: Interference and Inhibition in Bilingual Language Comprehension: Evidence from Polish-English Interlingual Homographs
Source: PLoS One. 2016 Mar 15;11(3):e0151430. doi: 10.1371/journal.pone.0151430 (PMC4792378; doi:10.1371/journal.pone.0151430)
Supplement: S3 Appendix — (PDF) [file pone.0151430.s003.pdf]

	First word in pair	Second word in pair
Block 1	office	dessert
	ship	ocean
Block 2	short	chalk
	orange	pear
Block 3	elephant	flour
	daisy	tulip
Block 4	bread	escape
	carrot	rabbit
Block 5	dirty	rope
	big	small
Block 6	violin	border
	amber	beach
Block 7	penguin	knife
	bell	church
Block 8	pepper	moon
	vote	democracy
Block 9	tank	chocolate
	bear	honey
Block 10	poet	stone
	angel	devil
Block 11	smith	hammer
	cereal	movie
Block 12	money	bank
	cherry	ear
